# Effectiveness of cognitive behaviour therapy for the treatment of catastrophisation in patients with fibromyalgia: a randomised controlled trial

**DOI:** 10.1186/ar3496

**Published:** 2011-10-23

**Authors:** Marta Alda, Juan V Luciano, Eva Andrés, Antoni Serrano-Blanco, Baltasar Rodero, Yolanda López del Hoyo, Miquel Roca, Sergio Moreno, Rosa Magallón, Javier García-Campayo

**Affiliations:** 1 Servicio de Psiquiatría, Hospital Miguel Servet y Universidad de Zaragoza, Instituto Aragonés de Ciencias de la Salud (I+CS), Red de Actividades Preventivas y de Promoción de la Salud (REDIAPP) (G06/170 and RD06/0018/0017), Avda Isabel La Catolica 1, 5009 Zaragoza, Spain; 2 Parc Sanitari Sant Joan de Déu, and Fundación Sant Joan de Déu, Red de Actividades Preventivas y de Promoción de la Salud (REDIAPP) (G06/170 and RD06/0018/0017), c/Dr Antoni Pujadas 40, 08830 Sant Boi de Llobregat, Barcelona, Spain; 3 Unidad Epidemiología Clínica, Hospital 12 de Octubre, CIBER Epidemiología y Salud Pública, Avda de Córdoba s/n, 28.041 Madrid, Spain; 4 Clínica de Neurociencias, Centro Rodero, c/Ruamayor 11, 39008 Santander, Spain; 5 Departamento de Psicología y Sociología, Universidad de Zaragoza, Instituto Aragonés de Ciencias de la Salud (I+CS), Red de Actividades Preventivas y de Promoción de la Salud (REDIAPP) (G06/170 and RD06/0018/0017), c/Pedro Cerbuna 12, 50009 Zaragoza, Spain; 6 Institut Universitari d'Investigació en Ciències de la Salut (IUNICS), University of Balearic Islands, Red de Actividades Preventivas y de Promoción de la Salud (REDIAPP) (G06/170 and RD06/0018/0017), c/Andrea Doria 55, 07014 Palma de Mallorca, Spain; 7 Centro de Salud Arrabal, Instituto Aragonés de Ciencias de la Salud (I+CS), Red de Actividades Preventivas y de Promoción de la Salud (REDIAPP) (G06/170 and RD06/0018/0017), Andador Aragües del Puerto 2-4, 50015 Zaragoza, Spain

**Keywords:** catastrophisation, fibromyalgia, randomised controlled trial, cognitive-behaviour therapy

## Abstract

**Introduction:**

No randomised, controlled trials have been conducted to date on the efficacy of psychological and pharmacological treatments of pain catastrophising (PC) in patients with fibromyalgia. Our aim in this study was to assess the effectiveness of cognitive-behaviour therapy (CBT) and the recommended pharmacological treatment (RPT) compared with treatment as usual (TAU) at the primary care level for the treatment of PC in fibromyalgia patients.

**Methods:**

We conducted a six-month, multicenter, randomized, blinded, parallel group, controlled trial in which patients were randomly assigned to one of three study arms: CBT (*n *= 57), RPT (*n *= 56) and TAU at the primary care level (*n *= 56). The major outcome of this study was PC in patients with fibromyalgia. The secondary variables were pain acceptance, depression, anxiety, pain, global function and quality of life.

**Results:**

CBT significantly decreased global PC at the six-month follow-up examination with effect sizes of Cohen's *d *= 0.73 and 1.01 compared with RPT and TAU, respectively. CBT was also more effective than RPT and TAU at increasing pain acceptance at the six-month follow-up examination (effect sizes of Cohen's *d *= 0.77 and 0.80, respectively). Compared with RPT and TAU, CBT was more effective at improving global function based on the Fibromyalgia Impact Questionnaire (six-month effect sizes Cohen's *d *= 0.44 and 0.53, respectively) and quality of life based on the European Quality of Life Scale (six-month effect sizes Cohen's *d *= 0.11 and 0.40, respectively). There were no differences among the three treatments with regard to pain and depression.

**Conclusions:**

CBT shows higher efficacy than RPT and TAU not only in key outcomes in FM, such as function and quality of life, but also in relevant mediators of treatment effects, such as pain catastrophising and pain acceptance.

**Trial registration:**

ISRCTN: ISRCTN10804772

## Introduction

The role of pain catastrophising (PC) in mediating responses to pain has received considerable attention in recent years [1-3], and a consistent relation between PC and distress reactions to painful stimulation has been demonstrated [3]. Although the defining criteria for PC have never been explicitly stated, there is general consensus that this construct involves an exaggerated negative orientation toward noxious stimuli. The aetiology of PC is not clear. It has been demonstrated that interpersonal mechanisms may not play a significant role in its development [4], whereas insecure attachment is positively associated with it [5]. Some of the consequences that have been associated with PC are more intense pain [6], heightened pain behaviour [7-9], greater analgesic consumption [10], reduced involvement in daily activities [3], occupational disability [11-13], suicidal ideation [14], increased use of healthcare services and longer hospital stays [15,16].

A positive association has been documented between depression and catastrophism [4], but this construct is different from the negative thoughts found in depression. Depressive thoughts are present only when associated with depressive mood; however, PC is considered a continuous psychological variable that is normally distributed even in healthy individuals without pain or depression [17]. The kinds of cognitions that characterise depression and catastrophism are also different: depressive thoughts are related to depression and similar concepts, such as inferiority, guilt or suicide. Catastrophising cognitions are exclusively focused on pain: a negative vision of it (magnification), continuously thinking about it (rumination) and the impossibility of controlling it (helplessness). A scale has been developed to measure PC: the Pain Catastrophizing Scale (PCS) [6].

Fibromyalgia (FM) is a prevalent and disabling disorder characterised by a history of widespread pain for at least three months and patient-reported tenderness in at least 11 of 18 defined tender points when digitally palpated with about 4 kg per unit area of force [18]. PC is a key risk factor of FM; in fact, PC is one of the most commonly used classifications to differentiate the clinical subtypes of FM [19]. PC occurs at higher rates in people with FM compared with other rheumatologic populations. Moreover, there is often an even stronger relationship between PC and key clinical outcomes, such as pain intensity and pain sensitivity, in comparison with other rheumatologic diseases [20-22].

However, despite the importance of PC, only one study of the psychological treatment of patients with PC has been conducted, and the only outcome assessed in that study was the general satisfaction of the patient and his or her knowledge about PC [23]. Our present study was not a randomised, controlled trial. According to a recent meta-analysis of the psychological interventions in FM [24], only five randomised, controlled trials have assessed PC as one of the outcomes [25-29]. To the best of our knowledge, there have been no studies of the pharmacological treatment of PC.

The aim of the present study was to assess the effectiveness of cognitive-behaviour therapy (CBT) and the recommended pharmacological treatment (RPT) for FM and to compare them with treatment as usual (TAU) at the primary care level for the treatment of PC in patients with FM. The secondary objective was to determine how depression, anxiety and pain contribute to predicting the response of PC to CBT.

## Materials and methods

### Design

We conducted a six-month, multicentre, randomised, parallel group, controlled trial in which patients were randomly assigned to one of three study arms (ratio 1:1:1): CBT, RPT with pregabalin and an antidepressant (duloxetine) if there was comorbid depression and (3) TAU at the primary care level. Evaluators were blinded to participants' treatment group assignments. The protocol of this study has been previously published [30]. This trial followed the Initiative on Methods, Measurement, and Pain Assessment in Clinical Trials, or IMMPACT, recommendations for chronic pain clinical trials [31] and the Consolidated Standards of Reporting Trials, or CONSORT, recommendations for randomised, controlled trials [32]. No changes to the methods were made after the trial began.

### Setting and study sample

Patients were recruited from any of the 41 primary healthcare centres in the city of Zaragoza, Spain. Zaragoza is the fifth-largest city in Spain, with a population of 713, 000. This study was carried out from January 2009 to June 2010. Participants were recruited from January to December 2009, and the six-month follow-up examinations were completed from January to June 2010. Patients were consecutively recruited by doctors working in primary care centres until the required sample size was attained, without a quota of patients assigned from each centre. Patients considered for inclusion were 18 to 65 years of age, able to understand and read Spanish, fulfilled the criteria for FM according to the American College of Rheumatology [18], had undergone no psychological treatment during the preceding two years, were receiving no pharmacological treatment at that time or were willing to discontinue it for two weeks before the start of the study, and had signed an informed consent statement. Those excluded were patients with severe axis I psychiatric disorders (dementia, schizophrenia, paranoid disorder and alcohol and/or drug abuse); patients with severe axis II psychiatric disorders or other medical disorders that, from the clinician's point of view, prevented the patient from following the treatment protocol; women who were pregnant or nursing; and those who declined to participate.

### Randomisation, treatment arms, implementation and masking of the study groups

#### Randomisation

Each patient was assigned to one of the three groups by a computer-generated random number sequence. Randomisation was stratified by the existence of comorbid depression to ensure a balance of patients with depression in the three groups.

#### Group assignment

The allocation sequence was generated by a member of the research group who was not involved in the study. Patients were automatically assigned to a group according to the random allocation sequence. The sequence was concealed until interventions were assigned. Patients agreed to participate before the random allocation and without knowing which treatment they would receive.

#### Implementation

The family doctors recruited the patients and assessed them for comorbidity of depression for stratification of the sample. They were unaware of the allocation sequence and were informed by telephone of the treatment group to which the patient was assigned. Central telephone assignment according to the computer-generated random allocation sequence was performed by a researcher with no clinical involvement in the trial. The recruiting doctor thus obtained each patient's group assignment instantly by telephone. RPT was administered by two psychiatrists (JGC and MA), TAU was administered by family doctors, and psychological interventions were delivered by trained therapists (SM and BR). Study personnel who conducted psychological assessments (RM and YLdH) were blinded to participants' treatment conditions. Owing to the characteristics of the trial, patients and therapists who administered any of the treatments were not blinded to the treatment that patients received.

### Intervention

#### Psychological intervention

We used a manual-based protocol derived from Thorn's model [33] that focuses on treating PC [34]. Our group adapted this model to treat people with FM [35]. This intervention was previously used and described in a pilot study of the treatment of PC in patients with FM [26], and its efficacy was assessed in a recent meta-analysis [24].

The CBT intervention mainly consists of two major components: cognitive restructuring, which focuses on reducing pain-specific dysfunctional cognitions (primarily PC), and coping, which focuses on teaching cognitive and behavioural coping strategies. In summary, this intervention encompasses ten weekly 90-minute CBT group sessions, including nine standard CBT sessions that are based on Thorn's program [33] and one specific session on PC (session 8). The duration of the intervention is 10 to 12 weeks. The program is structured as follows. Session 1: the connection between stress and pain. Session 2: identification of automated thoughts. Session 3: evaluation of automated thoughts. Session 4: questioning the automatic thoughts and constructing alternatives. Session 5: nuclear beliefs. Session 6: nuclear beliefs on pain. Session 7: changing coping mechanisms. Session 8: coping with ruminations, obsessions and worrying. Session 9: expressive writing. Session 10: assertive communication.

Session 8 is the additional PC session that begins after the coping session. This session is directed especially at participants who show high rumination. It consists of instructing the patients to write a story regarding the worst possible scenario for the future based on their greatest fear. This story should stress aspects that generate the greatest amount of malaise (for example, 'How do you see yourself in this situation?', 'What do you think?', 'How do you feel?', and so forth). The story is audiorecorded for a subsequent presentation to the patient. Patients are instructed to listen to this story for 30 to 60 minutes until it no longer causes anxiety. In general, this process takes between 10 and 15 sessions.

This treatment is highly structured and conducted in a group format with a maximum of eight patients per group. Because this psychotherapy program is strongly structured and patient participation is emphasised and focused on the task, the interactions among the patients are limited. These groups do not allow for the type of therapeutic interactions found in psychodynamic groups.

Trained therapists at the Torrero health centre administered the psychotherapy. Random sessions were audiorecorded and assessed by other members of the team to confirm that CBT techniques were exclusively used. Groups were consecutively created to fulfil the required sample size. The patients were occasionally allowed to use minor analgesics during the study, but not pregabalin, gabapentin, opioids or antidepressants.

#### Recommended pharmacological treatment

In 2007, the US Food and Drug Administration (FDA) approved pregabalin as the first drug to manage the symptoms of FM in the United States. Within 18 months, this agency also approved duloxetine and milnacipran for the same purpose. Although these drugs are marketed in Europe for other purposes, the European regulatory authorities recently rejected extending their approval of these drugs to include the treatment of FM [36]. On the basis of FDA recommendations and the Spanish Consensus for the Treatment of Fibromyalgia [37], treatment with pregabalin (300 to 600 mg/day) and duloxetine (60 to 120 mg/day) was administered to patients with major depressive disorder as diagnosed according to the Mini-International Neuropsychiatric Interview (MINI). A psychiatrist administered RPT and conducted follow-up with patients at baseline and each month after baseline during the six-month study.

#### Treatment as usual at the primary care level

The TAU group received the standard care offered by general practitioners at their health centres. To improve this group's treatment, the doctors received the 'Guide for the Treatment of Fibromyalgia in Primary Care' [38], which is edited and distributed by the Aragonese Health Service. 'Treatment as usual' implies that doctors selected a pharmacological treatment as well as the frequency of patient visits that they considered adequate. However, the treatment recommended in the guide that they received matched that of the recommended pharmacological intervention.

Neither the RPT patients nor the TAU patients received any psychological intervention during the six-month trial. The duration of RPT for both groups was the full six months.

### Measurements

The study personnel who carried out the measurements were kept blinded to which treatment each patient received. The assessments took place at baseline, posttreatment and after one, three and six months. Posttreatment assessment took place nine weeks after the baseline assessment for all groups, because this was the amount of time required for the CBT group to complete group therapy.

### Main outcome variables

The primary objective of this study was to assess the efficacy of CBT, RPT and TAU for the treatment of PC in patients with FM in primary care settings. The major outcome of this study was PC in patients with FM. This construct was assessed using the Spanish version [39] of the PCS [6]. The PCS is a 13-item self-report questionnaire that comprises three dimensions: rumination, magnification and helplessness. There is no established 'cutoff' point, because PC is considered to be distributed in a continuous way in the general population. All items are rated on a Likert scale from 0 (not at all) to 4 (all the time). The total possible score ranges from 0 to 52, with a higher score indicating higher PC.

### Secondary variables

The secondary objectives of this study were to evaluate, in patients with FM, the efficacy of CBT, RPT and TAU in primary care for depression (measured using the Hamilton Rating Scale for Depression (HAM-D)); anxiety (assessed using the Hamilton Anxiety Rating Scale (HARS)); pain (measured using the Pain Visual Analogue Scale (PVAS)); global function (assessed using the Fibromyalgia Impact Questionnaire (FIQ)); and quality of life (assessed using the European Quality of Life Scale 5-D (EuroQol-5D) Questionnaire).

#### Sociodemographic variables

The following patient data were collected: gender, age, marital status (single, married or in a relationship, separated or divorced, or widowed), ethnic group, living arrangements (alone, with spouse or partner, with offspring and/or spouse or partner, with other relatives, or with others), educational level (no formal education, primary school, secondary school, or university), employment status (unemployed, paid employment, on sick leave from paid employment, retired/pensioner or permanent disability), and income (measured using the minimum monthly salary in Spain).

#### Clinical variables

The clinical variables considered were years since the diagnosis of FM, preference for psychotherapy, comorbid depression, and sexual abuse and whether the patient was engaged in litigation at that time.

#### Psychiatric interview

Psychiatric disorders were diagnosed by conducting the MINI psychiatric interview [40], an instrument developed for use in primary care settings.

#### Hamilton Rating Scale for Depression

The HAM-D is probably the most used interview-based depressive symptom rating scale [41]. Although the original scale had 21 items, Hamilton suggested scoring only the initial 17 items because the last 4 items either occurred infrequently or described only aspects of the illness. Items are ranked on a scale of 0 to 4 (items with quantifiable severity) or 0 to 2 (items that measure symptoms that are more difficult to assess reliably). The greatest severity is indicated by a score of 2 or 4. The range for the 17-item scale is 0 to 50. The most used thresholds used are the following: very severe, > 23; severe, 19 to 22; moderate, 14 to 18; mild, 8 to 13; and normal, < 8 [42]. We used the validated Spanish version of HAM-D [43].

#### Hamilton Anxiety Rating Scale

The HARS is a clinician-administered rating scale that consists of 14 items [44]. Each item is rated on a 5-point scale (from 0 = no symptoms to 4 = severe, grossly disabling symptoms). Total scores for the HAS range from 0 to 56. A score of 14 or greater has been suggested to indicate clinically significant anxiety. We used the validated Spanish version of HARS [45].

#### Pain Visual Analogue Scale

PVAS records the subject's self-assessed pain on a Visual Analogue Scale (VAS), a 10-cm vertical line numbered from 0 to 100, with 0 representing no pain and 100 representing maximum pain [46].

#### Chronic Pain Acceptance Questionnaire

The Chronic Pain Acceptance Questionnaire (CPAQ) is a 20-item inventory designed to measure the patient's acceptance of pain [47]. CPAQ measures two principal factors: engagement in activities and pain willingness. All items are rated on a 0 (never true) to 6 (always true) scale. Nine items measuring pain willingness are reverse-keyed. The maximum possible total score is 120, with a higher score indicating better acceptance. The validated Spanish version of CPAQ was used [48].

#### Fibromyalgia Impact Questionnaire

The FIQ is a 10-item self-report questionnaire developed to measure the health status of FM patients [49]. The first item focuses on the patient's ability to carry out physical activities. In the next two items, patients are asked to circle the number of days in the past week during which they felt good and how often they missed work. Each of the last seven questions (job ability, pain, fatigue, morning tiredness, stiffness, anxiety and depression) is measured on a VAS. We used the translated and validated Spanish version of the FIQ [50].

#### EuroQoL-5D questionnaire (Spanish version)

The EuroQol-5D (EQ-5D) questionnaire is a generic instrument used to capture health-related quality of life [51]. It has two parts. Part 1 records patients' self-reported problems in each of five domains: mobility, self-care, usual activities, pain and/or discomfort and anxiety and/or depression. Each domain is divided into three levels of severity corresponding to no problems, some problems and extreme problems. Part 2 records the subject's self-assessed health on a VAS, a 10-cm vertical line on which the best and worst imaginable health states are scored 100 and 0, respectively.

### Statistical methods

#### Sample size

To calculate the sample size, it was necessary to know the effectiveness of pharmacological and psychological treatments on the main outcome variable: PC. There are no prior published studies on the pharmacological treatment of PC in FM. According to Glombiewski *et al*.'s meta-analysis [24], the effect size (using Hedges' *g*, a variation of Cohen's *d *that corrects for biases due to small sample sizes) of psychological interventions on PC in patients with FM ranges from 0.07 [12] to 1.9 [26]. Owing to this enormous variability, and on the basis of previous studies [25-30], we aimed to detect a difference of 25% or more between any of the groups (control and intervention). Accepting an α risk of 0.05 and *P *= 80% in a bilateral contrast, we needed 55 patients in each group [52]. Calculating 5% of refusals as found in previous studies [25-30], we needed a sample size of 58, which implies a total sample of 174 patients with FM.

#### Analysis strategy

All statistical analyses were performed using IBM SPSS Statistics version 19.0 software (IBM Corp, Armonk, NY, USA). First, we compared the sociodemographic and clinical characteristics of the three groups to verify that there were no significant differences among them at baseline. We used means ± SD for the continuous variables and percentages for the categorical variables. For comparisons, we used analysis of variance (ANOVA) for continuous variables (with *post hoc *Tukey's honestly significant difference test) and Χ^2 ^test with continuity corrections (or 2 × 2 Fisher's exact test when appropriate) for categorical variables.

In the present work, participants who provided a baseline and at least one posttreatment measurement comprised the intention-to-treat population. The outcomes were analysed using the last observation carried forward method. After the Kolmogorov-Smirnov test was performed to assess distributions for normality, analysis of covariance (ANCOVA) that included baseline scores as covariates was performed to examine the differences among the PC scale total scores of the three groups posttreatment and at the six-month follow-up examination for each of the PCS domains (rumination, magnification and helplessness) and secondary variables (CPAQ, HAM-D, HARS, PVAS, FIQ and EQ-5D). ANCOVA has greater statistical power than ANOVA to detect changes from baseline in randomised designs [53]. We selected the Bonferroni method to adjust the significance level of subsequent pairwise contrasts. The one- and three-month follow-up data were not analysed, because we focused only on the main assessment period (that is, posttreatment and the six-month follow-up).

The overall α level was set at 0.05. We also report the effect sizes (that is, the omnibus partial η_p_^2 ^value). In this case, η_p_^2 ^can be interpreted as the proportion of variance in the outcome that is attributable to each effect. The rule of thumb for η_p_^2 ^is that 0.01 is small, 0.06 is medium and 0.14 is large. Additionally, we computed Cohen's *d *for each pairwise comparison. The rule of thumb for Cohen's *d *is that 0.20 is small, 0.50 is medium and 0.80 is large.

### Ethical aspects of the study

This study followed Helsinki Convention norms and later modifications and the Declaration of Madrid of the World Psychiatric Association. The study protocol was approved by the Ethical Review Board of Aragon. All patients provided their written informed consent before the commencement of any study activities or procedures.

## Results

### Sample recruitment

**Figure 1:**
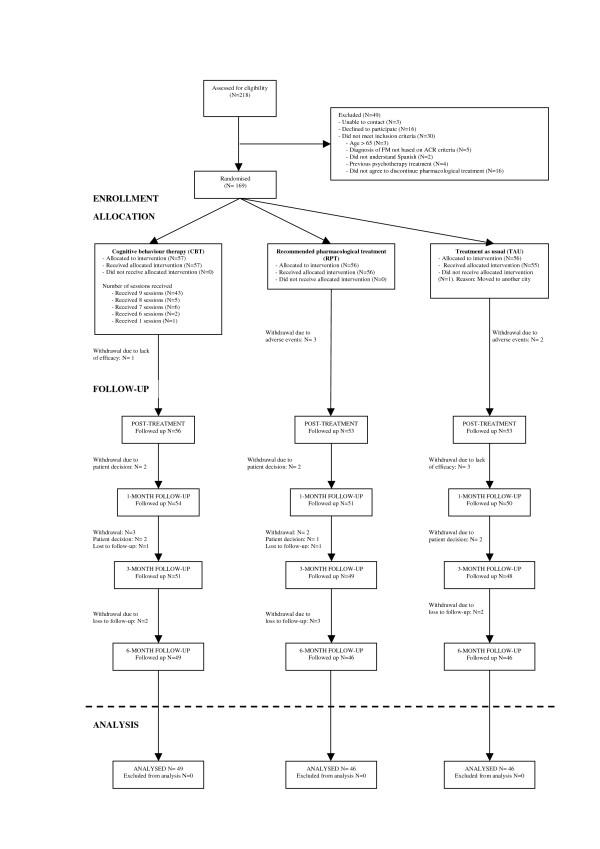
**Flowchart of the study**.

A total of 218 patients were screened, and 49 were excluded (Figure 1). Of these 49 patients, 30 were ineligible because they did not meet the entry criteria, 16 decided not to participate, 3 were impossible to contact and 169 were enrolled. Of the patients enrolled, 57 were randomly assigned to the CBT group, 56 to the RPT group and 56 to the TAU group. All of them received the allocated intervention (*N *= 168), except one patient in the TAU group who moved to another city during the study period. A total of 141 patients (83.9%) completed the study, comprising 49 (85.9%) in the CBT group, 46 (82.1%) in the RPT group and 46 (83.6%) in the TAU group (Figure 1). The patients' reasons for discontinuation were as follows. In the CBT group, one patient (1.7%) did not complete the study due to lack of efficacy of the treatment, four patients (7%) due to a personal decision and three patients (5.2%) were lost to follow-up. In the RPT group, three patients (5.3%) withdrew due to adverse effects of the treatment (two due to digestive problems and the other due to dizziness), three patients (5.3%) due to a personal decision and four patients (7.1%) were lost to follow-up. In the TAU group, two patients (3.6%) withdrew due to adverse events, two patients (3.6%) due to a personal decision, three patients due to lack of efficacy (5.4%) and two patients (3.6%) were lost to follow-up. The three groups did not differ significantly with regard to the percentage of patients who completed the study or to the reasons for discontinuation.

### Baseline demographic and clinical characteristics

The sample was mainly made up of middle-aged, married European females who lived with a spouse and/or offspring, had a primary or secondary school education, were unemployed or on sick leave and had a low or medium income level. From a clinical point of view, the patients, on average, had had FM for more than ten years. Half of them had been diagnosed with major depressive disorder, more than 10% had experienced sexual abuse and about 25% were currently engaged in litigation (Table 1). There were no statistically significant differences in baseline demographics or clinical characteristics between the three treatment groups, except for income, which was lower in the TAU group (Table 1). The questionnaire scores at baseline revealed that the participants had high PC, low pain acceptance, moderate depression, severe pain, limited global function and low quality of life (see Table 2). Moreover, the patients' anxiety levels were not clinically significant. There were significant differences (*P *< 0.05) between the groups with regard to acceptance and anxiety at baseline. *Post hoc *comparisons indicated that the CBT group had higher acceptance scores than the RPT and TAU groups (Cohen's *d *= 0.48 and 0.43, respectively). In addition, the RPT group had significantly higher anxiety than the TAU group (Cohen's *d *= 0.50).

**Table 1:** Baseline sociodemographic and clinical characteristics of patients with fibromyalgia by treatment group

Sociodemographic variables	Cognitive behaviour therapy group (*N *= 57)	RPT group (*N *= 56)	TAU group (*N *= 55)	Statistics
Females, *n *(%)	54 (94.7%)	52 (92.9%)	53 (96.4%)	X_2 _= 0.674
				*df *= 3
				*P *= 0.714
Mean age, years (± SD)	46.35 (6.71)	47.12 (6.25)	47.04 (6.53)	*F *= 0.240
				*df *= 165
				*P *= 0.787
Marital status, *n *(%)				X_2 _= 0.279
				*df *= 4
				*P *= 0.991
Married or in a relationship	40 (70.2%)	40 (71.4%)	37 (67.3%)	
Single	9 (15.8%)	9 (16.1%)	10 (18.2%)	
Separated or divorced	8 (14%)	7 (12.5%)	8 (14.5%)	
Widowed	0 (0%)	0 (0%)	0 (0%)	
Ethnic group, *n *(%)				*P *= 1
European	57 (100%)	57 (100%)	57 (100%)	
Living arrangement, *n *(%)				X_2 _= 1.481
				*df *= 8
				*P *= 0.993
Living alone	4 (7.0%)	4 (7.1%)	6 (10.9%)	
Living with spouse or partner	8 (14.0%)	9 (16.1%)	8 (14.5%)	
Living with offspring and/or spouse/partner	34 (59.6%)	30 (53.6%)	31 (56.4%)	
Living with other relatives	5 (8.8%)	7 (12.5%)	5 (9.1%)	
Other	6 (10.5%)	6 (10.7%)	5 (9.1%)	
Educational level, *n *(%)				X_2 _= 1.578
				*df *= 4
				*P *= 0.813
Illiterate	0 (0%)	0 (0%)	0 (0%)	
Primary school	23 (40.4%)	23 (41.1%)	28 (50.9%)	
Secondary school	23 (40.4%)	22 (39.3%)	18 (32.7%)	
University	11 (19.3%)	11 (19.6%)	9 (16.4%)	
Employment status, *n *(%)				X_2 _= 1.295
				*df *= 8
				*P *= 0.996
Unemployed	19 (29.8%)	15 (26.8%)	15 (27.3%)	
Paid employment	9 (15.8%)	11 (19.6%)	9 (16.4%)	
On sick leave from paid employment	13 (22.8%)	12 (21.4%)	14 (25.5%)	
Retired/pensioner	7 (12.3%)	6 (10.7%)	8 (14.5%)	
Permanent disability	11 (19.3%)	12 (21.4%)	9 (16.4%)	
Income				X_2 _= -10.04
				*df *= 4
				*P *= 0.40
< MS (600€/month)	15 (26.3%)	15 (26.8%)	27 (49.1%)	
1 to 2 MS	24 (42.1%)	23 (41.1%)	20 (36.4%)	
> 2 to 4 MS	18 (31.6%)	18 (32.1%)	8 (14.5%)	
> 4 MS	0 (0%)	0 (0%)	0 (0%)	
Clinical variables				
Mean years since diagnosis (± SD)	12.91 (7.15)	11.23 (3.85)	11.69 (4.02)	*F *= 1.544
				*df *= 165
				*P *= 0.217
Preference for psychotherapy, *n *(%)	28 (49.1%)	26 (46.4%)	27 (49.1%)	X_2 _= 0.107
				*df *= 2
				*P *= 0.948
Comorbid major depressive disorder, *n *(%)	27 (47.4%)	26 (46.4%)	30 (54.5%)	X_2 _= 0.874
				*df *= 2
				*P *= 0.646
Sexual abuse, *n *(%)	4 (7.0%)	7 (12.5%)	11 (14.5%)	X_2 _= 1.70
				*df *= 2
				*P *= -0.427
Currently engaged in litigation, *n *(%)	17 (29.8%)	12 (21.4%)	16 (29.1%)	X_2 _= 1.23
				*df *= 2
				*P *= 0.539

MS = minimum salary; RPT = recommended pharmacological treatment; TAU = treatment as usual group.

**Table 2:** Analyses of covariance (modified intention-to-treat analysis by last observation carried forward method) for mean scores on primary and secondary outcome measures by group at baseline, posttreatment and six-month follow-up

Outcome measures	Mean CBT (± SD)	Mean RPT (± SD)	Mean TAU (± SD)	*F*-value	*P*-value	η_p_^2^	Pairwise comparisons	Cohen's *d* CBT vs RPT	Cohen's *d* CBT vs TAU	Cohen's *d* RPT vs TAU
PCS total score (0 to 52)										
Baseline	34.13 (9.29)	32.19 (7.05)	31.23 (7.18)							
Posttreatment	24.79 (7.41)	31.36 (7.10)	31.47 (6.90)	133.34	0.001	0.63	1 < 2, 3	0.91	0.93	0.02
Six-month follow-up	25.50 (7.24)	30.64 (6.75)	32.74 (7.04)	144.33	0.001	0.65	1 < 2 < 3	0.73	1.01	0.30
PCS-Rumination (0 to 16)										
Baseline	11.87 (3.08)	11.08 (2.57)	10.92 (2.77)							
Posttreatment	8.82 (2.47)	10.68 (2.53)	11.09 (2.60)	100.82	0.001	0.56	1 < 2, 3	0.74	0.90	0.16
Six-month follow-up	9.02 (2.46)	10.28 (2.50)	11.34 (2.61)	87.82	0.001	0.53	1 < 2 < 3	0.51	0.91	0.42
PCS-Magnification (0 to 12)										
Baseline	6.38 (2.63)	6.23 (2.40)	5.92 (2.22)							
Posttreatment	5.18 (2.27)	6.15 (2.48)	6.19 (2.23)	33.08	0.001	0.30	1 < 2, 3	0.41	0.45	0.02
Six-month follow-up	5.59 (2.32)	6.34 (2.42)	6.62 (2.27)	23.65	0.001	0.23	1 < 2 < 3	0.32	0.45	0.12
PCS-Helplessness (0 to 24)										
Baseline	15.89 (5.07)	14.89 (3.98)	14.57 (4.13)							
Posttreatment	10.79 (4.04)	14.55 (3.98)	14.36 (3.99)	74.29	0.001	0.49	1 < 2, 3	0.94	0.90	0.05
Six-month follow-up	10.95 (4.01)	14.02 (3.85)	14.94 (4.27)	68.04	0.001	0.46	1 < 2 < 3	0.78	0.96	0.23
CPAQ (0 to 120)										
Baseline	49.00 (10.33)	44.40 (8.90)	44.45 (10.80)							
Posttreatment	51.30 (9.53)	43.36 (9.00)	43.15 (10.86)	33.07	0.001	0.29	1 > 2, 3	0.86	0.80	0.02
Six-month follow-up	50.46 (9.37)	43.47 (8.85)	42.53 (10.40)	24.97	0.001	0.24	1 > 2, 3	0.77	0.80	0.10
HAM-D (0 to 50)										
Baseline	14.47 (3.93)	14.94 (4.03)	14.09 (4.64)							
Posttreatment	7.78 (2.46)	7.98 (1.80)	8.17 (2.25)	2.17	0.12	0.03	ns	0.09	0.17	0.09
Six-month follow-up	7.91 (2.50)	8.19 (1.96)	8.57 (2.47)	4.05	0.02	0.05	1 < 3	0.12	0.27	0.17
HARS (0 to 56)										
Baseline	10.84 (4.27)	11.22 (3.75)	9.50 (2.98)							
Posttreatment	7.09 (2.96)	7.11 (2.39)	7.40 (2.18)	9.71	0.001	0.11	1, 2 < 3	0.01	0.12	0.13
Six-month follow-up	7.25 (3.02)	7.39 (2.57)	7.58 (2.07)	8.49	0.001	0.10	1, 2 < 3	0.05	0.13	0.08
PVAS (0 to 100)										
Baseline	64.20 (10.78)	68.13 (9.84)	64.72 (10.44)							
Posttreatment	36.88 (8.29)	37.14 (10.53)	38.68 (7.48)	2.25	0.109	0.03	ns	0.03	0.23	0.17
Six-month follow-up	40.68 (10.93)	40.54 (9.61)	44.34 (8.56)	7.48	0.001	0.09	2 < 3	0.01	0.37	0.42
FIQ (0 to 100)										
Baseline	65.91 (10.85)	66.36 (9.88)	64.48 (10.50)							
Posttreatment	46.21 (9.18)	50.93 (9.38)	48.64 (6.77)	6.96	0.001	0.08	1 < 2, 3	0.51	0.30	0.28
Six-month follow-up	48.80 (9.11)	52.84 (9.17)	53.26 (7.54)	11.22	0.001	0.12	1 < 2, 3	0.44	0.53	0.05
EuroQol VAS (0 to 100)										
Baseline	44.55 (16.47)	46.82 (15.62)	43.87 (14.50)							
Posttreatment	60.45 (16.63)	58.00 (13.07)	53.49 (14.40)	11.49	0.001	0.13	1 > 2, 3	0.16	0.45	0.33
Six-month follow-up	58.39 (16.27)	56.73 (13.85)	52.26 (14.03)	10.44	0.001	0.12	1 > 2, 3	0.11	0.40	0.32

CBT = cognitive-behaviour therapy; CPAQ = Chronic Pain Acceptance Questionnaire; EuroQol VAS = EuroQol Visual Analogue Scale; FIQ = Fibromyalgia Impact Questionnaire; HAM-D = Hamilton Rating Scale for Depression; HARS = Hamilton Anxiety Rating Scale; PCS = Pain Catastrophizing Scale; PVAS = Pain Visual Analogue Scale; RPT = recommended pharmacological treatment; TAU = treatment as usual. ns = nonsignificant difference between groups (*P *> 0.05).

### Effectiveness in the main outcome: pain catastrophising

Table 2 displays the means ± SD for all outcome variables at baseline, posttreatment and the six-month follow-up. As shown in the right column, the ANCOVA yielded significant effects for global PC and the three PCS dimensions (rumination, magnification and helplessness). The pairwise comparisons yielded the same pattern of results for all PCS dimensions. After treatment, we found that CBT had been more effective than the other two treatments in reducing PC, rumination, magnification and helplessness. These improvements were still observed at the six-month follow-up. In addition, we found that RPT was more effective than TAU at reducing all of the PCS dimensions.

### Effectiveness in secondary outcomes

ANCOVA revealed significant effects (see Table 2) of pain acceptance (CPAQ), depression (HAM-D), anxiety (HARS), pain (PVAS), functional impairment (FIQ) and health-related quality of life (EuroQol VAS). Pairwise comparisons within the pain acceptance findings indicated that the patients who received CBT had improved more at the posttreatment and six-month follow-up examinations than those assigned to other treatments. There were no significant differences between the groups with regard to depression posttreatment; however, we found that depression had reduced more in the CBT group than in the TAU group at the six-month follow-up. CBT and RPT were equally effective at reducing anxiety: Both treatment options were significantly better than TAU posttreatment and at the six-month follow-up. There were no significant differences in pain level between the groups posttreatment. Pain levels had reduced more for the RPT group than for the TAU group by the six-month follow-up. An analysis of functional impairment revealed a group effect that favoured the CBT group posttreatment and at the six-month follow-up. We observed a group effect of the EuroQol VAS, such that patients who received CBT showed more improvement than those treated with RPT or TAU posttreatment and at the six-month follow-up.

## Discussion

### Characteristics of the study

To the best of our knowledge, this is the first study to assess the efficacy of CBT, RPT and TAU in reducing PC in patients diagnosed with FM. Only five previous randomised, controlled trials assessed the efficacy of treatments on PC in FM patients [26-30], but none of them considered PC as the main outcome. Other strengths of this study are the assessment of the influences of variables such as depression, anxiety or pain on the variations in PC. These results can be generalised because of the high external validity of the sample study (recruited in primary care settings). One of the main limitations of the study is the number of secondary variables analysed, which raises the concern of obtaining a significant result by chance (type I error). However, because of the lack of similar studies and the range of possible outcomes in FM [30], we considered it appropriate to use several different outcome measures.

### Treatment efficacy in terms of the main outcome: pain catastrophising

CBT significantly decreased global PC at the six-month follow-up, with effect sizes of Cohen's *d *= 0.73 and 1.01 compared with RPT and TAU, respectively. Previous studies in which the effectiveness of CBT in reducing PC in FM patients was assessed found effect sizes ranging from 0.17 [29] and 0.26 [25] up to a maximum of 0.56 [29]. The effect sizes in our study are similar to those found in previous studies on the efficacy of CBT in FM patients and in a meta-analysis of psychological and pharmacological treatments for FM administered at primary and secondary levels of healthcare [30]. However, our current study is the first to assess the effectiveness of CBT compared with RPT and TAU on reducing the three subscales of PC. It confirms that CBT was significantly more effective than the other two treatments.

In some previous studies, comparison of CBT with placebo therapies for the treatment of PC in FM patients produced inconsistent results. For instance, in Vlaeyen *et al*.'s study [29], CBT was less effective (effect size 0.17) than education (effect size 0.27). In our study, we found that CBT was more effective than both RPT and TAU and that RPT was more effective than TAU in the reduction of all PCS dimensions.

### Effectiveness in terms of the secondary outcomes

CBT is more effective than RPT or TAU, both at posttreatment and at the six-month follow-up, for the following secondary outcomes measured in this study: pain acceptance, anxiety, global function and quality of life. However, CBT is not more effective than the other two interventions at reducing depression and pain.

CBT was more effective than RPT and TAU at increasing pain acceptance at the six-month follow-up (effect sizes of Cohen's *d *= 0.77 and 0.80, respectively). This report is one of the first to study the effectiveness of CBT in treating pain acceptance, a key concept of third-wave therapies, especially Acceptation and Commitment Therapy (ACT) [54]. ACT proposes that attempting to control internal events such as pain sensations and negative emotional reactions is problematic. 'Pain acceptance' is a psychological construct that refers to the process of learning to live with pain. Although we have described the components of the psychotherapy, we did not include ACT elements. We hypothesise that CBT increases pain acceptance because it promotes the acquisition of diverse skills needed to manage pain. For instance, cognitive restructuring enhances coping (by reframing) and might affect pain acceptance. Indeed, a previous study found great similarities between a behavioural coping strategy (that is, task persistence) and pain acceptance [55].

Regarding depression, there are no differences among the three treatments in decreasing depression either posttreatment or at the six-month follow-up. This is not surprising, because patients in the pharmacological and TAU groups who were diagnosed with depression on the basis of the MINI psychiatric interview used antidepressants according to treatment guidelines [37,38]. Many studies have demonstrated that pharmacological and psychological treatments are similarly effective in treating depression [30]. Researchers in most previous studies of CBT in FM patients have found effect sizes of about 0.3 to 0.4, with some outliers reaching 1.22 [25]. Some investigators have found CBT to be ineffective for treating depression in FM patients [29]. Another meta-analysis concluded that the efficacy of CBT in treating depression could not be distinguished without some risk of bias [56]. In our study, CBT was effective for decreasing depression, but RPT and TAU were also quite effective, so we found no significant differences among the three.

In our study, CBT and RPT improved anxiety symptoms significantly more than did TAU. All patients in the pharmacological group were treated with pregabalin, a drug that is effective for both pain and anxiety. In addition, about 50% of patients in this group who were diagnosed with depression took antidepressants that are also effective for anxiety. We do not think this is due to low effectiveness of CBT, because previous studies of anxiety have confirmed that it is effective [57]. Instead, we believe that this result was obtained because TAU is effective in reducing anxiety. It is difficult to compare these results with those of previous studies, because anxiety is not usually an outcome of interest for psychological interventions in FM patients [25,30].

In our study, CBT did not improve pain (as assessed by PVAS) more than the other two treatments either posttreatment or at the six-month follow-up. Only RPT is more effective than TAU at six months. Previous studies of CBT in FM patients [24] have found effect sizes of 0.35 to 0.5 for pain. In our study, all of the patients in the RPT arm took pregabalin, a potent analgesic, and over half of them also took duloxetine, which, in addition to being an antidepressant, has an analgesic effect. It seems that for pain, CBT cannot overcome the effect of RPT. The lesser effectiveness of TAU can be attributed to many family doctors' not systematically using pregabalin to treat FM patients or duloxetine to treat patients with associated depression.

For patients with FM, CBT, compared with RPT and TAU, was more effective at improving global function as assessed by the FIQ (six-month effect sizes Cohen's *d *= 0.44 and 0.53, respectively) and quality of life as assessed by the EuroQol VAS (six-month effect sizes: Cohen's *d *= 0.11 and 0.40, respectively). Previous studies of CBT in FM patients [24] have found effect sizes of 0.2 to 0.5 based on the FIQ [24]. However, a recent meta-analysis in which quality of life was assessed [56] suggested that CBT is not effective for the achievement of this outcome. CBT may be more effective than other treatments at changing these two variables because they are global assessments of the patient, and CBT is more able than pharmacological approaches to improve many aspects of FM.

How pharmacotherapy might improve PC has not been discussed herein, thus only tentative suggestions can be proposed to explain our results. The current conceptualisations of pain incorporate a biopsychosocial approach that involves behavioural reactions (for example, avoidance behaviour), cognitive reactions (for example, PC) and physiological reactions (for example, elevated autonomic arousal and muscle tension). These approaches are highly related and establish a vicious cycle [58]. RPT improves behavioural and physiological reactions [59], thus their effects might indirectly affect cognitive reactions (that is, PC).

## Conclusions

Our present study confirms that PC, an important outcome in patients with FM [24-29], significantly improved after CBT compared with RPT or TAU. CBT also improved other relevant outcomes, such as pain acceptance, anxiety, global function and quality of life. On the basis of the results of our study, and from a clinical point of view, we can recommend that clinicians systematically include CBT in the management of patients with FM. It may not be so advisable to include CBT in the treatment of FM from a cost-effectiveness point of view, as researchers in some meta-analyses have found [56]. In fact, investigators in some meta-analyses [30] did not observe differences between standard primary care treatments and more specialised approaches delivered at the secondary level. In any case, new randomised, controlled trials with larger samples are necessary to definitively decide the role of CBT in the standard care of patients with FM.

Despite the overall high efficacy of CBT, new research should focus on improving this efficacy even more. Some interesting future directions include the early detection and treatment of patients who are at risk of developing FM [60], considering 'stage of chronicity' as a moderator of vulnerability [48] and subdividing FM patients according to their distinctive, contextual cognitive-behavioural patterns [19].

## Abbreviations

ACT: acceptance and commitment therapy; ANCOVA: analysis of covariance; ANOVA: analysis of variance; CBT: cognitive-behaviour therapy; CPAQ: Chronic Pain Acceptance Questionnaire; EQ-5D: EuroQol 5-D Questionnaire; EuroQoL: European Quality of Life Scale; FIQ: Fibromyalgia Impact Questionnaire; FM: fibromyalgia; HAM-D: Hamilton Rating Scale for Depression; HARS: Hamilton Anxiety Rating Scale; ITT: intention to treat; PC: pain catastrophising; PCS: Pain Catastrophising Scale; RPT: recommended pharmacological treatment; VAS: Visual Analogue Scale; TAU: treatment as usual.

## Competing interests

The authors declare that they have no competing interests.

## Authors' contributions

MA, JGC, PS, BR, ASB, RM and MR conceived the study design. MA, SM and YLdH collected the data, EA, JVL and BR conducted the statistical analysis. All authors contributed to the interpretation of the results and the drafting of the manuscript, and all authors approved the final manuscript for publications.
